# Glucosamine exposure reduces proteoglycan synthesis in primary human endothelial cells *in vitro*

**DOI:** 10.3402/fnr.v60.32615

**Published:** 2016-09-23

**Authors:** Trine M. Reine, Trond Geir Jenssen, Svein Olav Kolset

**Affiliations:** 1 Department of Nutrition, Institute of Basic Medical Sciences, University of Oslo, Oslo, Norway; 2 Section of Nephrology, Department of Transplant Medicine, Oslo University Hospital, Rikshospitalet, Oslo, Norway; 3 Metabolic and Renal Research Group, Faculty of Health Sciences, UiT The Arctic University of Norway, Tromsø, Norway

**Keywords:** arthritis, endothelial cells, glucosamine, glycosaminoglycan, proteoglycan

## Abstract

**Purpose:**

Glucosamine (GlcN) supplements are promoted for medical reasons, for example, for patients with arthritis and other joint-related diseases. Oral intake of GlcN is followed by uptake in the intestine, transport in the circulation and thereafter delivery to chondrocytes. Here, it is postulated to have an effect on synthesis and turnover of extracellular matrix constituents expressed by these cells. Following uptake in the intestine, serum levels are transiently increased, and the endothelium is exposed to increased levels of GlcN. We investigated the possible effects of GlcN on synthesis of proteoglycans (PGs), an important matrix component, in primary human endothelial cells.

**Methods:**

Primary human endothelial cells were cultured *in vitro* in medium with 5 mM glucose and 0–10 mM GlcN. PGs were recovered and analysed by western blotting, or by SDS-PAGE, gel chromatography or ion-exchange chromatography of ^35^S-PGs after ^35^S-sulphate labelling of the cells.

**Results:**

The synthesis and secretion of ^35^S-PGs from cultured endothelial cells were reduced in a dose- and time-dependent manner after exposure to GlcN. PGs are substituted with sulphated glycosaminoglycan (GAG) chains, vital for PG function. The reduction in ^35^S-PGs was not related to an effect on GAG chain length, number or sulphation, but rather to the total expression of PGs.

**Conclusion:**

Exposure of endothelial cells to GlcN leads to a general decrease in ^35^S-PG synthesis. These results suggest that exposure to high levels of GlcN can lead to decreased matrix synthesis, contrary to what has been claimed by supporters of such supplements.

Use of glucosamine (GlcN) as a dietary supplement for patients with arthritis has been highly debated (1, 2). Articular cartilage is composed of chondrocytes embedded in an extracellular matrix mainly composed of collagens and proteoglycans (PGs) (3). In arthritis, cartilage erosion is a prominent clinical feature. GlcN supplements have been used with the intention to increase the supply of the building blocks for PG biosynthesis, to increase synthesis and to decrease degradation of cartilage. However, it is highly controversial whether dietary supply of GlcN and chondroitin is of any use in this disease (4, 5). It is not documented that arthritis is in any way related to lack of nutrients. The data provided so far have not provided any documentation of molecular mechanisms for a positive effect of GlcN on cartilage *de novo* biosynthesis. Arthritis is an inflammatory disease, and GlcN is also suggested to be beneficial for these patients through anti-inflammatory effects (6).

Plasma levels of GlcN are normally very low, and GlcN is synthesised from glucose in each cell. However, GlcN can also be taken up by cells through glucose transporters. GlcN is further converted to uridine diphosphate (UDP)-N-Acetyl glucosamine (UDP-GlcNAc) and UDP-N-Acetylgalactosamine (UDP-GalNAc), building blocks for the glycosaminoglycan (GAG) side chains of PGs.

Articular cartilage does not have blood vessels, nerves or lymphatics, and delivery of circulatory molecules is slow. Intake of GlcN supplements is most likely not able to increase the GlcN availability in cartilage to any great extent. The endothelial cells, however, are lining cells of the blood vessels and are regularly exposed to high levels of GlcN after ingestion. Consequently, how endothelial cells respond to elevated levels of GlcN is a relevant nutritional issue. For this reason, the aim of this study was to investigate the effects of increased GlcN levels on primary human endothelial cells (HUVEC). The results presented here show that endothelial cells respond to increased GlcN levels by decreasing the synthesis and secretion of PGs. This is the opposite of what is hypothesised for chondrocytes to give therapeutic beneficial effects.

## Materials and methods

### Primary human endothelial cells

HUVEC were isolated from infant umbilical vein of delivering mothers at Rikshospitalet University Hospital (7). None of the mothers providing umbilical cords had any pregnancy complications, and ethical approval for the use of HUVEC was obtained from the Human Research Ethics Committee.

The cells were established in MCDB 131 medium (Sigma) containing 5 mM glucose and supplemented with 7% heat inactivated foetal calf serum (FCS, Sigma), basic fibroblast growth factor (1 ng/ml, R&D), epidermal growth factor (10 ng/ml, R&D), hydrocortisone (1 µg/ml, Sigma), gentamicin (50 µg/ml, GIBCO Invitrogen) and fungizone (250 ng/ml, GIBCO Invitrogen). Cells were used for experiments within three passages, and culture medium was changed every 48–72 h. For experiments, the cells were exposed to GlcN (0.1, 1, 2, 5 or 10 mM), and medium with physiologic glucose concentration (5 mM) was used as a control in all experiments. If not otherwise mentioned, each result presented is a representative of three experiments on HUVEC from different donors.

### Cell viability

The possible cytotoxic effects of GlcN were investigated by measurement of lactate dehydrogenase (LDH) activity released from damaged cells using the Cytotoxicity Detection Kit (Roche) according to the manufacturer's instructions.

To determine the effects of GlcN on HUVEC proliferation and cell viability, the adherent cells were released by trypsin treatment and counted in a haemocytometer (Bürker, Germany) after the addition of 0.4% trypan blue. Cells positive for trypan blue were categorised as dead cells.

### ^35^S-macromolecules

*De novo* synthesised PGs are readily detected by metabolic labelling of their highly sulphated GAG chains by the addition of ^35^S-sulphate (Hartmann Analytic) to the culturing medium. In order to increase labelling efficiency, the medium was changed to sulphate-free RPMI 1640 medium (GIBCO Invitrogen) containing 5 mM glucose and 5 mM L-glutamine (Sigma) and FCS reduced to 2%. HUVEC were labelled with 0.1–0.2 mCi/ml ^35^S-sulphate for 24 h. During this period, the cells were also exposed to GlcN (D-(+)-GlcN hydrochloride, Sigma) of different concentrations indicated in the individual experiments.

After complete labelling, the medium fractions were harvested and centrifuged to remove cell debris. Cells were carefully rinsed in ice cold phosphate buffered saline (PBS) and solubilised in 4 M guanidine hydrochloride with 2% Triton X-100 in 0.05 M sodium acetate buffer pH 6.0. In order to isolate the macromolecules and remove excess unincorporated ^35^S-sulphate, 1 ml of each fraction was applied to 4 ml columns of Sephadex G-50 Fine (GE Healthcare) gel chromatography in 0.05 M Tris-HCl pH 8.0 with 0.05 M NaCl. The first 1 ml eluate was discarded, and the following 1.5 ml eluate, containing the ^35^S-macromolecules, was collected. The amount of ^35^S-macromolecules was determined by scintillation counting of aliquots of the material before further analysis. Cell fraction protein was determined using the Uptima BC Assay Protein Quantification Kit (Interchim). Prior to analysis by gel filtration, ion-exchange chromatography and, in some cases, SDS-PAGE, the anionic ^35^S-PGs were further purified from the ^35^S-macromolecules by diethylaminoethanol (DEAE) ion-exchange chromatography. Samples were applied to individual DEAE-sephacel poly-prep columns equilibrated in 0.15 M NaCl Tris-buffer (0.05 M Tris-HCl pH 8.0), washed in 0.15 M NaCl Tris-buffer and 0.3 M NaCl Tris-buffer before eluted in 2 M NaCl Tris-buffer.

### SDS-PAGE

^35^S-macromolecules from HUVEC were analysed by SDS-PAGE on precast 4–20% gradient gels (BioRad). ^14^C-labelled rainbow standards (Amersham) were used for molecular weight determinations. Samples were desalted and concentrated using Microcon Centrifugal Filter Devices (Millipore) and vacuum centrifugation and then run on SDS-PAGE, either untreated or after degradation of GAG chains. For degradation of ^35^S-chondroitin sulphate (CS) and dermatan sulphate (DS), samples from media (~5,000 cpm per analysis) were incubated at 37°C overnight with 0.01 units of chondroitinase ABC (cABC, E.C. 4.2.2.4, Sigma Aldrich) in 0.05 M Tris-HCl pH 8.0, containing 0.05 M sodium acetate. In parallel samples, heparan sulfate (HS) was depolymerised by HNO_2_ deamination at pH 1.5, cleaving the polysaccharide at N-sulphated GlcN units as described (8). In some experiments, aliquots were subjected to cABC treatment prior to HNO_2_ treatment, in order to depolymerise both the HS and the CS/DS-GAGs present.

Prior to loading, the samples were incubated at room temperature for 15 min in sample buffer with 1% SDS and mercaptoethanol as reducing agent. After complete electrophoresis, the gels were treated with fixing solution (isopropanol 25%, glacial acetic acid 10%), amplified (Amersham), dried on an SGD 2000 slab gel dryer (Thermo Savant), subjected to autoradiography for 3–7 days in −70°C using Amersham Hyperfilm™ ECL (GE Healthcare) and finally visualised using an AGFA Curix-60 developer.

### Western blotting

Confluent HUVEC were exposed to 1 mM GlcN for 24 h in standard culturing MCDB medium with FCS reduced to 2%. Medium fractions were harvested and centrifuged to remove cell debris. Cells were rinsed in ice cold PBS and solubilised in radioimmunoprecipitation Assay (RIPA) buffer (50 mM Tris-HCl pH 7.5, 150 mM NaCl, 1% Triton X-100, 1% SDS, 1% Na-deoxycholate, 10 mM ethylenediaminetetraacetic acid (EDTA), 10 mM Na_4_PO_2_O_7_ and Complete Mini EDTA-free cocktail tablet from Roche). ^35^S-PGs from all fractions were purified by DEAE ion-exchange chromatography and concentrated using Microcon Centrifugal Filter Devices (Millipore) or vacuum centrifugation. HS and CS/DS-GAGs in media collected from HUVEC in the presence or absence of 1 mM GlcN were depolymerised using cABC or HNO_2_ as described.

For determination of the presence of perlecan or decorin in the different media, the samples obtained were separated on SDS-PAGE and electroblotted to a polyvinyl difluoride (PVDF) membrane (Millipore, USA) using the Criterion™gel system (BioRad). Primary antibodies against rabbit anti-human perlecan, kindly provided by prof. RV Iozzo (1:500 dilution), and goat anti-human decorin (1:4,000, R&D systems) were used. The secondary antibody used was HRP-linked donkey anti-rabbit IgG (1:50,000, GE Healthcare) and rabbit anti-goat IgG (1:1,000, R&D systems), respectively. The membranes were developed using ECL western blotting detection reagents (GE Healthcare) and finally exposed to film and developed using the AGFA Curix-60 developer. For molecular weight determination, standards were run simultaneously in separate lanes (BioRad).

### Gene expression analysis

RNA was isolated from HUVEC using the E.Z.N.A Total RNA Kit 1 according to the manufacturer's instructions and reverse transcribed using the High Capacity RNA-to-cDNA Kit (Applied Biosystems). Quantitative real-time PCR (qRT-PCR) was performed on an ABI Prism 7900HT (Applied Biosystems) using TaqMan Gene Expression Master Mix and the predesigned TaqMan Gene Expression Assays with RPL30 as endogenous control (*HSPG2*, perlecan, Hs00194179_m1, *SRGN* serglycin Hs010041559_m1, *SDC4* syndecan-4 Hs00161617_m1, *BGN* biglycan Hs00156076_m1 and *RPL30* 60S ribosomal protein L30 Hs00265497_m1). The relative mRNA levels for each transcript were calculated using the ΔΔCt method.

### Macromolecular properties of ^35^S-PGs

Both secreted and cell-associated ^35^S-PGs were isolated by G50 fine gel chromatography and DEAE ion-exchange chromatography. The samples were separated according to size on a Sepharose CL-4B column, either untreated or as ^35^S-CS/DSPGs after HNO_2_ treatment or ^35^S-HSPGs after cABC treatment. The column was run in 0.05 M Tris-HCl pH 8 with 0.2 M NaCl, and fractions of 400–800 µl were collected and analysed for radioactivity by scintillation counting. The elution profiles were determined relative to the elution of the V_0_ marker dextran blue (2,500 kDa) and the V_t_ marker phenol red (0.36 kDa).

NaOH treatment leads to the release of intact GAG chains from their core proteins by β-elimination. ^35^S-macromolecules from conditioned medium and cell lysate were either left untreated or subjected to HS depolymerisation by HNO_2_ treatment or CS/DS digestion by cABC treatment. The resulting CS/DS and HSPGs were subjected to alkali β-elimination for release of the intact GAGs from their core proteins. Samples containing CS/DS and/or HS GAGs were recovered by DEAE ion-exchange chromatography and concentrated and desalted on vivaspin ultrafiltration devices (molecular weight cut-off 30 kDa, GE Healthcare). The samples were further separated according to size on a Sepharose CL-6B column run in 0.05 M Tris-HCl pH 8 with 0.2 M NaCl. Fractions were collected and analysed for radioactivity by scintillation counting. The elution profiles were determined relative to the elution of the V_0_ marker dextran blue (2,500 kDa) and the V_t_ marker phenol red (0.36 kDa).

### Anionic properties of ^35^S-PGs

^35^S-macromolecules recovered from medium were subjected to cABC or HNO_2_ treatment. The resulting ^35^S-HSPGs or ^35^S-CS/DS PGs were isolated by DEAE ion-exchange chromatography. After dilution or salt removal on vivaspin ultrafiltration devices, the samples were analysed by ion-exchange chromatography with a salt gradient ranging from 0.15 to 2 M NaCl in 0.05 M Tris-HCl pH 8. Aliquots of each fraction were subjected to scintillation counting and determination of the elution profile of the internal standard CS-6 (Sigma) by the 1,9 dimethyl methylene blue assay.

### Statistical analysis

The data were analysed using GraphPad Prism 5.03. A Wilcoxon's matched-pairs signed rank test was run to determine if there were differences in cpm. The different GlcN concentrations were compared to 0 mM GlcN, and the different time-points were compared to baseline (0 h). Differences of *p*≤0.05 were considered significant.

## Results

**Fig. 1:**
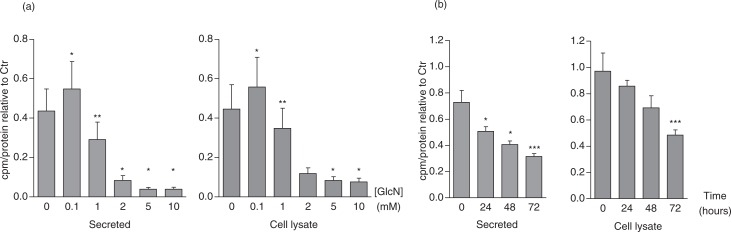
(a) HUVEC were exposed to the indicated GlcN concentrations and metabolically labelled for 24 h. (b) Cells exposed to 1 mM GlcN for the indicated times were metabolically labelled for the last 24 h. All responses, both for medium (left) and cell lysate (right), are given as the cpm/protein ratios expressed relative to the control (0 mM GlcN). The difference compared to control (ctr) was tested, and differences of *p*≤0.05 were considered significant. *p<0.05, **p<0.01, ***p<0.001.

HUVEC were exposed to different concentrations of GlcN ranging from 0.1 to 10 mM and labelled with ^35^S-sulphate for 24 h. Radiolabelled macromolecules were recovered by gel chromatography. ^35^S-macromolecules in HUVEC are almost exclusively comprised of ^35^S-PGs (9). From Fig. 1a, it is evident that there is a dose-dependent decrease in both the synthesis and release of ^35^S-PGs from HUVEC after incubation with GlcN, suggesting that both synthesis and secretion of ^35^S-PGs were reduced by GlcN. The protein content was measured in parallel cell cultures, and there was no difference between the different HUVEC cultures, irrespective of GlcN concentrations used. No cytotoxic effects of GlcN were found using the LDH assay. In addition, cells were counted after trypsin treatment for detachment of the cells. The trypan blue exclusion test showed high viability and correlation between cell number and protein content up to GlcN concentrations of 1 mM. At concentrations above 1 mM, cell number was slightly reduced. For this reason, 1 mM GlcN was used in all further experiments.

HUVEC were then exposed to 1 mM GlcN for different time periods, and the amount of ^35^S-PGs was determined in the cell and medium fractions. A time-dependent decrease in the amount of ^35^S-PGs was observed both in the medium and in the cell fractions (Fig. 1b), supporting that both synthesis and secretion were inhibited in HUVEC cultured in the presence of GlcN.

**Fig. 2:**
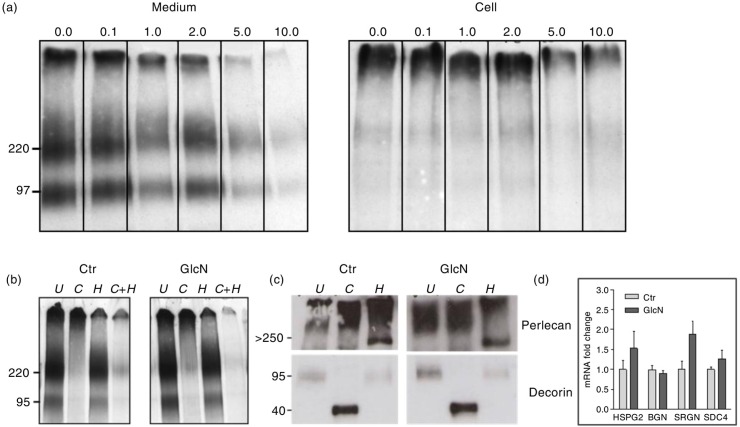
(a) HUVEC were exposed to the indicated concentrations of GlcN and metabolically labelled for 24 h. The size and relative expression of the ^35^S-PGs obtained from medium (left panel) and cell lysate (right panel) are visualised by subjection to SDS-PAGE after protein standardisation. (b) HUVEC cultured in 5 mM glucose alone (ctr) or added 1 mM GlcN were metabolically labelled for 24 h. Samples of isolated ^35^S-PGs were standardised to cpm and subjected to either cABC digestion (*C*), HNO_2_ depolymerisation (*H*), a combination of the above (*C*+*H*), or left untreated (*U*). The size and GAG substitution of ^35^S-PGs secreted from ctr and GlcN-stimulated cells are apparent after subjection to SDS-PAGE. (c). HUVEC were cultured in 5 mM glucose (ctr) or 1 mM GlcN for 24 h. The level of secretion and the GAG substitution of the PGs perlecan and decorin are evident after subjection to western blotting. The isolated PGs were subjected to cABC digestion (*C*), HNO_2_ depolymerisation (*H*), a combination of the above (*C*+*H*), or left untreated (*U*) and protein content were standardised. The migration positions of high molecular weight markers are shown on the left side of the panel (in kDa). (d) Gene expression of perlecan (*HSPG2*), biglycan (*BGN*), serglycin (*SRGN*) and syndecan-4 (*SDC4*) in GlcN-treated cells was determined by qRT-PCR. Values are expressed as the fold change in GlcN-treated cells relative to control cells.

These findings were further corroborated by analysing the ^35^S-PGs by SDS-PAGE. The secreted ^35^S-PGs were clearly separated into three populations of almost equal intensity – one of high molecular weight (migrating at the top of the gel), a second with an approximate molecular weight of 220 kDa and a third species with a molecular weight around 100 kDa (Fig. 2a, left panel). This is in accordance with previous studies (10). The cell fraction, in contrast, mainly contained the high molecular weight component (Fig. 2a, right panel). In both fractions, the amount of these ^35^S-PGs decreased when the cells were exposed to increasing concentrations of GlcN. It is also evident from Fig. 2a that there was no prominent difference in PG sizes between PGs synthesised after exposure to increasing concentrations of GlcN. From these experiments, we conclude that treatment of HUVEC with GlcN resulted in a general decrease in *de novo* ^35^S-PG expression.

The decrease in PG expression could be the result of a decreased expression of either CS/DS-chains or HS-chains, the major PG species expressed by HUVEC (11). To analyse this, medium samples from control and GlcN-treated cells, containing equal amounts of cpm, were purified by Sephadex G50 fine and DEAE ion-exchange chromatography and subjected to SDS-PAGE after cABC degradation of CS/DS or HNO_2_ degradation of HS. From Fig. 2b, it is evident that the high molecular weight band on top of the gel is HSPGs, as it is degraded with HNO_2_ (H), both in medium from control and GlcN-treated cells. The ^35^S-PGs at molecular weights of approximately 220 kDa and 100 kDa were both CS/DS PGs, as can be seen by their susceptibility to cABC treatment (labelled C in both panels). A combination of both the treatments (C+H) revealed that almost all the labelled material was ^35^S-HSPGs and ^35^S-CS/DS PGs. These results suggest that GlcN treatment leads to a general decrease in ^35^S-PG biosynthesis and does not have a unique effect on the expression of neither CS nor HS-type GAG chains.

Each different HS and CS/DS band most likely contains several different PGs. These different PGs might have different functions and responses to GlcN. We have demonstrated that the extracellular matrix PGs perlecan and decorin are highly expressed in HUVEC (10). Western blotting was performed on untreated, cABC treated and HNO_2_-treated medium fractions with antibodies against the CS/DS PG decorin and the HSPG perlecan. Both the intact PGs and the core proteins of perlecan and decorin (Fig. 2c) were detected in the medium fractions of control and GlcN-treated HUVEC. The molecular weight of both intact perlecan and perlecan core protein was above 250 kDa. The decorin core protein had a molecular weight around 40 kDa, whereas intact decorin was found at 100 kDa after SDS-PAGE. Both PGs were expressed and secreted in both control and GlcN exposed HUVEC and the HS and CS/DS substitution was not affected. Accordingly, both control and GlcN-treated HUVEC express both decorin and perlecan, supporting the notion that the decrease in PG expression after GlcN treatment is a general effect rather than a selective effect on particular PGs. Furthermore, gene expression of basement membrane PG perlecan, the small leucine-rich PG biglycan, the cell surface PG syndecan-4 and the intracellular PG serglycin was determined, and none of these were significantly altered by GlcN treatment (Fig. 2d).

**Fig. 3:**
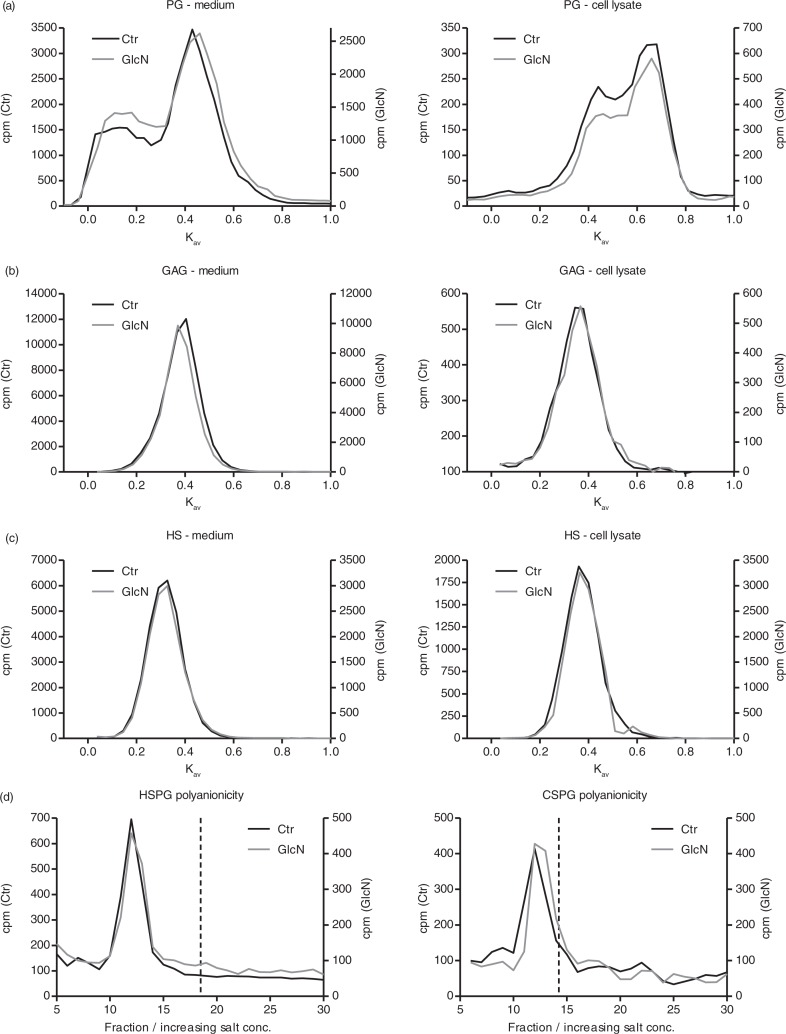
Both control and GlcN (1 mM) treated cells were metabolically labelled with ^35^S-sulphate for 24 h. (a) The ^35^S- PGs were isolated from the medium (left panel) and cell lysate (right panel) and analysed by Sepharose CL-4B chromatography. (b) The ^35^S-GAGs were obtained from the secreted (left panel) and cell-associated (right panel) PGs and analysed by Sepharose CL-6B chromatography. (c) ^35^S-HS were obtained from the secreted (left panel) and cell-associated (right panel) PGs and analysed by Sepharose CL-6B chromatography. These are one representative chromatogram out of three individual experiments. (d) The charge distribution of secreted ^35^S-HSPGs (left panel) or ^35^S-CSPGs (right panel) were determined by DEAE ion-exchange chromatography with a salt gradient ranging from 0.15 to 2.0 M NaCl. The peak fraction of the internal standard CS-6 is indicated by the dotted line. These results are based on one individual experiment. All responses, given in cpm, are scaled for convenience.

PGs are substituted with GAG chains of varying numbers, length and identity. Through these chains, the PGs interact with a wide range of partner molecules including matrix molecules, cytokines, growth factors and proteases. These chains are further modified by sulphation at distinct positions. Both the specific sulphation pattern and the overall negative charge created can be important for interaction with partner molecules. In summary, these chains are crucial for the functions of the PGs, and they can be modified by a changing environment. Here, we explored if GlcN exposure could modulate the structure of the GAG chains. First, the size distribution of ^35^S-PGs synthesised and secreted in the absence or presence of GlcN was compared. ^35^S-PGs were purified by ion-exchange chromatography and subjected to size determination by Sepharose CL-4B gel chromatography. Exposure to GlcN did not alter the size of neither the cell-associated nor the secreted ^35^S-PGs, evident from Fig. 3a, left and right panels, indicating no apparent effect on GAG substitution.

However, the observed lack of effect on PG size could be explained by simultaneous changes in GAG size and number. To analyse this in further detail, the ^35^S-PGs were subjected to alkali β-elimination. The resulting free ^35^S-GAGs were recovered by DEAE ion-exchange chromatography, and the effect of GlcN on the GAG chain size distribution was determined by Sepharose CL-6B gel chromatography. From Fig. 3b, it is evident that the overall chain length was not affected by exposure to GlcN.

To explore the effect of GlcN on the ^35^S-GAG components in further detail, ^35^S-PGs synthesised in the presence or absence of GlcN were subjected to enzymatic CS/DS-depolymerisation by cABC. The remaining ^35^S-HS were released from the core protein by alkali β-elimination. These HS-GAG chains were recovered by DEAE ion-exchange chromatography, and their relative length was determined by Sepharose CL-6B gel chromatography, as illustrated in Fig. 3c. The HS-GAG chain length was not affected by exposure to GlcN, supporting the observation in Fig. 2a.

The fact that neither PG size nor GAG chain length was affected by GlcN treatment suggests that the observed reduction in ^35^S-PGs is not caused by a reduction in neither HS nor CD/DS GAG chain length nor by the number of GAG chains substituted on the core proteins. However, the observed decrease in ^35^S-PGs could be caused by reduced sulphation of the GAG chains. For this reason, the effect of GlcN on the polyanionic properties of secreted HSPGs and CS/DSPGs was investigated using ion-exchange chromatography, see Fig. 3d. No differences in elution profiles were observed between samples from control or GlcN-stimulated cells, suggesting that there were no differences in the polyanionic properties of neither HSPGs nor CS/DS PGs after the GlcN treatment compared to controls. Accordingly, these data show that the decrease in ^35^S-PGs after GlcN exposure was not due to differences in polyanionic properties, for example, decreased sulphation.

Taken together, the reduction in ^35^S-PGs observed as an effect of GlcN treatment of primary HUVEC was not caused by a reduction in chain type, length, number or charge. The data presented suggest that incubating HUVEC *in vitro* with GlcN leads to a general decrease in the expression of the PGs.

## Discussion

PGs are important constituents of the extracellular matrix and are proteins modified with linear GAG chains. These chains are further modified by sulphate at distinct positions. Through the GAG chains, PGs have the ability to bind to partner molecules including matrix components, chemokines, growth factors and proteases, and thus, they offer structure as well as protection, transport and presentation of these molecules. Consequently, the structure of the GAG chains is decisive for the function of the PG (12).

In this study, we found that HUVEC exposed to increasing extracellular concentrations of GlcN decreased the synthesis and secretion of ^35^S-PGs in a dose- and time-dependent manner (Fig. 1). We investigated whether the GlcNAc-containing ^35^S-HS was more affected than the GalNAc-containing species ^35^S-CS/DS. By using cABC, which specifically degrades CS/DS and HNO_2_, pH 1.5, which only degrades HS and heparin, we show that there was no difference in the effects on these two different ^35^S-GAG types when HUVEC were incubated with GlcN (Fig. 2). Additionally, from Fig. 2, it is evident that the GAG substitution of HSPG perlecan and CS/DS PG decorin was not affected by GlcN. From these observations, we conclude that GlcN exposure does not affect the ratio between HS and CS/DS.

The reduction in ^35^S-sulphate incorporation seen in response to GlcN could be caused by a number of factors. A reduction in the synthesis of PG core protein, the number of GAGs attached to each core protein, the GAG chain length or finally the sulphation of the chains could all potentially explain this decrease. Studies in arterial smooth muscle cells show a reduction in PG size caused by reduced GAG chain length with increasing GlcN concentrations (13). However, our Sepharose CL-4B data showed that PG size was not affected by GlcN in HUVEC (Fig. 3a). To investigate the effect of GlcN on the GAG components, HS from PGs synthesised in the presence or absence of GlcN were isolated and separately subjected to chain length determination by Sepharose CL-6B chromatography, with no effect on chain length (Fig. 3b and c). Finally, no differences in polyanionicity were detected by ion-exchange chromatography of HS- and CS/DS PGs (Fig. 3d).

Together, these results show that the observed reduction in ^35^S-PGs is explained not by a reduction in HS or CS/DS ratio, chain length or chain number, nor by a reduction in the sulphation of the GAGs, but rather by a general decrease in the total expression of PGs. This is in line with what we have previously shown for HUVEC exposed to hyperglycaemic conditions, by incubating the cells in 25 mM glucose. Under these conditions, we also observed a decrease in PG expression and secretion (9). The results presented here show that HUVEC respond to GlcN exposure in the same manner by decreasing PG synthesis and secretion.

GAG chains are composed of repeating disaccharide units of uronic acid and hexosamine. The hexosamine may be either GlcN in HS and heparin or GalN in CS and DS. During biosynthesis, GlcN can be converted to UDP-GlcNAc or UDP-GalNAc, precursors of GAG synthesis. GlcN is therefore essential for the biosynthesis of PGs (14). Increased GlcN levels are thought to increase the flux through the hexosamine biosynthetic pathway; thus, increased levels of GlcN would be expected to increase the PG biosynthesis through increased availability of the substrate for GAG synthesis, the UDP-sugars. Alternatively, increased flux could affect PG levels by changes in the cytosolic turnover or transport into the Golgi lumen. However, an inhibition of incorporation of GlcN into macromolecules has been demonstrated after incubations with high concentrations of GlcN (15). This was correlated with a reduction in UDP-GlcNAc caused by ATP depletion by phosphorylation of GlcN into GlcN-6-phosphate (16–18). However, the GlcN concentrations used in those experiments were as high as 32 mM, and the experiments were performed in adipocytes and vascular smooth muscle cells. Also, in the muscle cells, GlcN exposure led to a decrease in ^35^S-sulphate incorporation, caused by shorter GAG chains (13).

Increased flux through the hexosamine pathway could also affect signalling pathways through O-GlcNAcylation of signalling proteins or transcription factors (19–21). Such changes may affect the regulation of PG biosynthesis. O-GlcNAc modifications have been found to be increased in the cartilage of patients with knee osteoarthritis, possibly caused by a proinflammatory milieu (22).

Some studies suggest that GlcN exerts most of its functions through suppression of inflammatory pathways, particularly nuclear factor-κB (NF-κB) signalling, and a decline in proinflammatory cytokines and enzymes (6). In contrast, others report a GlcN-induced inflammatory response in HepG2 cells with increased release of proinflammatory cytokines and NO together with insulin resistance (23).

The question whether dietary supplements containing GlcN or a combination of GlcN and chondroitin are effective treatments for patients with arthritis is still controversial (1, 2, 24–26). Articular cartilage has very limited ability to regenerate. The most prominent hypothesis is that PG biosynthesis in chondrocytes is stimulated as a result of increased GlcN supply, and a lower degradation of cartilage occurs, which leads to fewer symptoms in, for example, the knee (2). The availability in chondrocytes of dietary GlcN supplements has been questioned. Studies on cells and cartilage tissues have shown that the concentration of GlcN needed to stimulate PG expression is 10 to 1,000 fold higher than the transient concentration determined in serum or plasma a few hours after GlcN ingestion. Some reports suggest an increased aggrecan core protein formation (27), inhibition of aggrecan degradation (28) and reduction in cartilage degradation (29) in cultured chondrocytes exposed to high concentrations of GlcN *in vitro*. The maximum level of GlcN in serum was found to be in the range of 1.9–11.5 µM after oral administration of GlcN (30) or in another study, 40–60 µM in plasma (31). In bovine primary chondrocytes, physiological relevant concentrations of GlcN did not increase the intracellular levels of UDP-hexosamine. On the contrary, 1 mM GlcN seemed to increase the level of UDP-hexosamine, but this was not accompanied by an increase in ^35^S-sulphate incorporation or aggrecan mRNA level (32).

In this study, we have chosen to investigate the effects of GlcN on primary endothelial cells. *In vivo*, these cells are exposed to elevated levels of GlcN after oral intake of GlcN supplements. This has not been documented for chondrocytes, where the positive effects of GlcN supplements are assumed to take place. Our results suggest that if, in endothelial cells, GlcN has any effects on extracellular matrix constituents, such as PGs, it would be to decrease the biosynthesis rather than to increase it. However, whether these results can be extended also to be valid for chondrocytes is not known at present. Some studies on GlcN in animal models of arthritis have been performed. Interestingly, in a rat liver fibrosis model, GlcN decreased matrix accumulation (33). In another study, an effect of GlcN on cartilage trough effects on the liver is suggested. (34). This highlights the necessity to further investigate what role GlcN plays in other organs that contribute to inflammation, such as the endothelium. PGs constitute a diverse group of molecules with several different functions in vascular health and disease, not only as part of extracellular matrices structurally but also as part of inflammation and signal transduction (35). Thus, in endothelium, a reduced PG synthesis could affect endothelial function in different ways.

Our data do not support the notion that oral intake of GlcN supplements promotes matrix build-up. The alleged positive effects of GlcN, if any, could potentially be because of effects not related to GlcN itself, but rather to other bioactive components in these products. The presence of such components could be explained by the high binding capacity of the negatively charged GAGs and their degradation products including GlcN.

## References

[1] Clegg DO, Reda DJ, Harris CL, Klein MA, O'Dell JR, Hooper MM (2006). Glucosamine, chondroitin sulfate, and the two in combination for painful knee osteoarthritis. N Engl J Med.

[2] Sawitzke AD, Shi H, Finco MF, Dunlop DD, Bingham CO, Harris CL (2008). The effect of glucosamine and/or chondroitin sulfate on the progression of knee osteoarthritis: a report from the glucosamine/chondroitin arthritis intervention trial. Arthritis Rheum.

[3] Sophia Fox AJ, Bedi A, Rodeo SA (2009). The basic science of articular cartilage: structure, composition, and function. Sports Health.

[4] Silbert JE (2009). Dietary glucosamine under question. Glycobiol.

[5] Henrotin Y, Mobasheri A, Marty M (2012). Is there any scientific evidence for the use of glucosamine in the management of human osteoarthritis?. Arthritis Res Ther.

[6] Dalirfardouei R, Karimi G, Jamialahmadi K (2016). Molecular mechanisms and biomedical applications of glucosamine as a potential multifunctional therapeutic agent. Life Sci.

[7] Jaffe EA, Nachman RL, Becker CG, Minick CR (1973). Culture of human endothelial cells derived from umbilical veins. Identification by morphologic and immunologic criteria. J Clin Invest.

[8] Shively JE, Conrad HE (1976). Formation of anhydrosugars in the chemical depolymerization of heparin. Biochem.

[9] Gharagozlian S, Borrebaek J, Henriksen T, Omsland TK, Shegarfi H, Kolset SO (2006). Effect of hyperglycemic condition on proteoglycan secretion in cultured human endothelial cells. Eur J Nutr.

[10] Reine TM, Vuong TT, Rutkovskiy A, Meen AJ, Vaage J, Jenssen TG (2015). Serglycin in quiescent and proliferating primary endothelial cells. PLoS One.

[11] 
Meen AJ, Oynebraten I, Reine TM, Duelli A, Svennevig K, Pejler G (2011). Serglycin is a major proteoglycan in polarized human endothelial cells and is implicated in the secretion of the chemokine GROalpha/CXCL1. J Biol Chem.

[12] Varki AC, Cummings RD, Esko JD, Freeze H, Hart G, Marth J (1999). Essentials of glycobiology.

[13] Tannock LR, Little PJ, Wight TN, Chait A (2002). Arterial smooth muscle cell proteoglycans synthesized in the presence of glucosamine demonstrate reduced binding to LDL. J Lipid Res.

[14] Kjellén L, Lindahl U (1991). Proteoglycans: structures and interactions. Annu Rev Biochem.

[15] Plagemann PG, Erbe J (1973). Transport and metabolism of glucosamine by cultured Novikoff rat hepatoma cells and effects on nucleotide pools. Cancer Res.

[16] Marshall S, Nadeau O, Yamasaki K (2004). Dynamic actions of glucose and glucosamine on hexosamine biosynthesis in isolated adipocytes: differential effects on glucosamine 6-phosphate, UDP-N-acetylglucosamine, and ATP levels. J Biol Chem.

[17] Hresko RC, Heimberg H, Chi MM, Mueckler M (1998). Glucosamine-induced insulin resistance in 3T3-L1 adipocytes is caused by depletion of intracellular ATP. J Biol Chem.

[18] Little PJ, Drennon KD, Tannock LR (2008). Glucosamine inhibits the synthesis of glycosaminoglycan chains on vascular smooth muscle cell proteoglycans by depletion of ATP. Arch Physiol Biochem.

[19] Copeland RJ, Bullen JW, Hart GW (2008). Cross-talk between GlcNAcylation and phosphorylation: roles in insulin resistance and glucose toxicity. Am J Physiol Endocrinol Metab.

[20] Issad T, Kuo M (2008). O-GlcNAc modification of transcription factors, glucose sensing and glucotoxicity. Trends Endocrinol Metab.

[21] Love DC, Hanover JA The hexosamine signaling pathway: deciphering the ‘O-GlcNAc code’. Sci STKE 2005.

[22] Tardio L, Andres-Bergos J, Zachara NE, Larranaga-Vera A, Rodriguez-Villar C, Herrero-Beaumont G (2014). O-linked N-acetylglucosamine (O-GlcNAc) protein modification is increased in the cartilage of patients with knee osteoarthritis. Osteoarthritis Cartilage.

[23] Zhu D, Wang Y, Du Q, Liu Z, Liu X (2015). Cichoric acid reverses insulin resistance and suppresses inflammatory responses in the glucosamine-induced HepG2 cells. J Agricultural Food Chem.

[24] National Center for Complimentary and Alternative Medicine (2008). The NIH glucosamine/chondroitin arthritis intervention trial (GAIT). J Pain Palliat Care Pharmacother.

[25] Hochberg MC, Martel-Pelletier J, Monfort J, Moller I, Castillo JR, Arden N (2016). Combined chondroitin sulfate and glucosamine for painful knee osteoarthritis: a multicentre, randomised, double-blind, non-inferiority trial versus celecoxib. Ann Rheum Dis.

[26] McAlindon TE, LaValley MP, Gulin JP, Felson DT (2000). Glucosamine and chondroitin for treatment of osteoarthritis: a systematic quality assessment and meta-analysis. JAMA.

[27] Dodge GR, Jimenez SA (2003). Glucosamine sulfate modulates the levels of aggrecan and matrix metalloproteinase-3 synthesized by cultured human osteoarthritis articular chondrocytes. Osteoarthritis Cartilage.

[28] Sandy JD, Gamett D, Thompson V, Verscharen C (1998). Chondrocyte-mediated catabolism of aggrecan: aggrecanase-dependent cleavage induced by interleukin-1 or retinoic acid can be inhibited by glucosamine. Biochem J.

[29] Fenton JI, Chlebek-Brown KA, Peters TL, Caron JP, Orth MW (2000). Glucosamine HCl reduces equine articular cartilage degradation in explant culture. Osteoarthritis Cartilage.

[30] Biggee BA, Blinn CM, McAlindon TE, Nuite M, Silbert JE (2006). Low levels of human serum glucosamine after ingestion of glucosamine sulphate relative to capability for peripheral effectiveness. Ann Rheum Dis.

[31] Jackson CG, Plaas AH, Sandy JD, Hua C, Kim-Rolands S, Barnhill JG (2010). The human pharmacokinetics of oral ingestion of glucosamine and chondroitin sulfate taken separately or in combination. Osteoarthritis Cartilage.

[32] Qu CJ, Jauhiainen M, Auriola S, Helminen HJ, Lammi MJ (2007). Effects of glucosamine sulfate on intracellular UDP-hexosamine and UDP-glucuronic acid levels in bovine primary chondrocytes. Osteoarthritis Cartilage.

[33] Donia T, Ali EMM, Abdel-Hady Mostafa A-H (2016). Biochemical effects of glucosamine on glycoproteins of rats with hepatic fibrosis. Int J Adv Res.

[34] Panicker S, Borgia J, Fhied C, Mikecz K, Oegema TR (2009). Oral glucosamine modulates the response of the liver and lymphocytes of the mesenteric lymph nodes in a papain-induced model of joint damage and repair. Osteoarthritis Cartilage.

[35] Bonnans C, Chou J, Werb Z (2014). Remodelling the extracellular matrix in development and disease. Nat Rev Mol Cell Biol.

